# Documented risk-based antithrombotic management and 30-day outcomes after endoscopic hemostasis for non-variceal upper gastrointestinal bleeding: a retrospective cohort study

**DOI:** 10.3389/fmed.2026.1890670

**Published:** 2026-08-25

**Authors:** Xiaoyun Zhao, Li Wang, Jianyi Li

**Affiliations:** 1 Department of Gastroenterology, The Second People’s Hospital of Linfen, Linfen, Shanxi, China; 2 Endoscopy, Xixian County People’s Hospital, Xixian, Shanxi, China; 3 Department of Gastroenterology, Linfen Central Hospital, Linfen, Shanxi, China

**Keywords:** antithrombotic therapy, endoscopic hemostasis, non-variceal upper gastrointestinal bleeding, rebleeding, retrospective cohort, risk assessment, thromboembolism

## Abstract

**Objective:**

To evaluate whether documented adherence to a prespecified risk-based antithrombotic management framework was associated with 30-day rebleeding, thromboembolism, and net adverse clinical events (NACE) after therapeutic endoscopy for non-variceal upper gastrointestinal bleeding (NVUGIB).

**Methods:**

This single-center retrospective cohort included 150 unique index admissions from January 2021 through December 2025. Documented strategy adherence was coded in 100 patients and non-adherence in 50. Rebleeding was evaluated only after successful initial hemostasis (*n* = 141). Parsimonious Firth penalized logistic regression was used for the primary analyses. Thirty multiple imputations by chained equations with predictive mean matching and propensity-score overlap weighting were used as sensitivity analyses. Resumption timing was analyzed descriptively because temporal ordering did not support causal modeling.

**Results:**

Thirty-day rebleeding occurred in 13/141 patients (9.2%), thromboembolism in 16/150 (10.7%), death in 6/150 (4.0%), and NACE in 29/150 (19.3%). Rebleeding was observed in 5/96 adherent patients (5.2%) and 8/45 non-adherent patients (17.8%; Fisher *p* = 0.026); NACE occurred in 12/100 (12.0%) and 17/50 (34.0%), respectively (*p* = 0.002). Documented adherence was associated with lower rebleeding (adjusted odds ratio [aOR] 0.03, 95% CI 0.002–0.43) and NACE (aOR 0.04, 95% CI 0.007–0.20). The corresponding adjusted risk differences were −15.7 percentage points (95% CI −23.1 to −9.0) and −27.4 percentage points (95% CI −34.2 to −17.1). Multiple-imputation and overlap-weighted analyses were directionally concordant. Seven of 13 rebleeding events occurred on or before the recorded resumption date.

**Conclusion:**

Documented adherence to a risk-based management framework was associated with fewer 30-day adverse events. Because exposure classification depended on clinical documentation, residual confounding and misclassification remain likely, and the large adjusted effects should not be interpreted causally. Individualized multidisciplinary assessment appears more important than any single predefined resumption threshold.

## 1 Introduction

Non-variceal upper gastrointestinal bleeding (NVUGIB) represents a critical and common medical emergency encountered globally in acute care settings. Managing NVUGIB in patients receiving underlying antithrombotic therapy presents a delicate clinical tightrope: clinicians must rapidly achieve endoscopic hemostasis and secure mucosal healing while simultaneously limiting systemic thromboembolic complications caused by the temporary interruption or reversal of essential antiplatelet or anticoagulant medications. Contemporary practice guidelines consistently advocate early patient risk stratification, urgent diagnostic and therapeutic endoscopy, and individualized, indication-specific decisions regarding drug interruption and resumption, rather than applying a rigid, single universal restart interval (1–3).

Because acute gastrointestinal hemorrhage frequently occurs in multimorbid patients with complex cardiovascular, cerebrovascular, or renal comorbidities, modern care extends far beyond isolated endoscopic interventions. Effective management requires seamless, multidisciplinary coordination among emergency physicians, gastroenterologists, endoscopists, cardiologists, neurologists, hematologists, surgeons, and interventional radiologists (4, 5). Furthermore, holistic analyses of global scientific outputs highlight that gastrointestinal bleeding has increasingly evolved into a complex, cross-specialty clinical domain requiring structured health-system pathways to optimize patient-centered acute outcomes and reduce long-term morbidity (6). Real-world healthcare delivery thus relies heavily on integrated institutional communication to bridge emergency resuscitation, acute inpatient hemostasis, and post-discharge antithrombotic planning.

Despite clear international consensus recommendations, considerable clinical variation persists in real-world practice regarding the interruption, reversal, and timing of antithrombotic restart. The majority of published observational studies to date have evaluated outcomes by focusing primarily on isolated exposure variables, such as specific antithrombotic drug classes (e.g., direct oral anticoagulants versus vitamin K antagonists or antiplatelet agents) or fixed calendar intervals following index hemostasis (e.g., restarting within 3, 7, or 14 days) (7–9). While these isolated variables provide important mechanistic benchmarks, they frequently fail to capture the real-world complexity of clinical decision-making.

In everyday practice, a structured management pathway depends not merely on a single calendar cutoff, but on whether a comprehensive, multi-step risk assessment was systematically performed and documented. Such a structured process requires explicitly evaluating baseline bleeding severity, initial endoscopic stigmata, patient-specific thrombotic indications, temporary drug withholding or reversal choices, and a prospective, multidisciplinary resumption plan. In routinely collected electronic medical records, a holistic proxy such as ‘documented strategy adherence’ captures both the clinical fidelity of management and the quality of clinical documentation. This distinction is paramount because comprehensive documentation often correlates directly with clinician experience, timely multidisciplinary access, standardized nursing follow-up intensity, and the overall administrative quality of acute care.

Addressing whether adherence to a structured, multidisciplinary risk-based framework improves clinical endpoints remains an important research question. Understanding the short-term impact of structured peri-endoscopic antithrombotic management is particularly crucial given the recognized burden of early adverse events, emergency department revisits, and unplanned readmissions within 30 days after acute gastrointestinal bleeding episodes (10). Highlighting structural documentation and risk stratification may provide clinicians and health systems with actionable pathways to improve care delivery beyond static restart protocols.

Therefore, this study was designed to evaluate whether documented adherence to a prespecified risk-based antithrombotic management framework was associated with 30-day recurrent bleeding, thromboembolic events, and net adverse clinical events (NACE) among adult patients with NVUGIB who underwent therapeutic endoscopy. Resumption timing was systematically recorded and analyzed as a secondary descriptive process variable rather than as a causal substitute for the primary strategy adherence exposure. The overarching goal of this study is explicitly associative, transparent, and hypothesis-generating, providing pragmatic insights into quality-driven antithrombotic management in acute NVUGIB care.

## 2 Methods

### 2.1 Study design, setting, and participants

We conducted a single-center retrospective cohort study using routinely collected electronic medical records, endoscopy records, laboratory data, and 30-day follow-up records at The Second People’s Hospital of Linfen. The corrected analytic dataset contained admissions from January 2021 through December 2025. The study is reported with reference to STROBE and the RECORD extension (11, 12).

Eligible patients were aged at least 18 years, had NVUGIB while receiving antiplatelet or anticoagulant therapy before admission, underwent therapeutic endoscopy within 48 h of admission, and had evaluable exposure and 30-day outcome data. In the final cohort, 147/150 underwent endoscopy within 24 h; the median time was 12.4 h (range 1.4–26.6). Exclusions were variceal bleeding, no therapeutic endoscopy, no pre-admission antithrombotic exposure, unavailable exposure or primary-outcome data, and duplicate or recurrent admissions.

The index admission was defined objectively as the first eligible admission for a patient during the study period. Readmissions within 30 days were tracked as outcomes and were not counted as new independent subjects. A later episode could be considered independent only after a 30-day washout. The final analytic file contained 150 unique medical-record numbers. A total of 320 consecutive patient encounters with suspected upper gastrointestinal bleeding were screened for eligibility. Of these, 170 encounters were excluded based on prespecified exclusion criteria: variceal bleeding (*n* = 42), lack of pre-admission antithrombotic therapy (*n* = 58), diagnostic endoscopy only without therapeutic endoscopic hemostasis (*n* = 35), time to endoscopy exceeding 48 h after admission (*n* = 12), duplicate or recurrent admissions within the 30-day index washout window (*n* = 15), and incomplete medical records or loss to 30-day follow-up (*n* = 8). The final analytic cohort comprised 150 unique index admissions.

### 2.2 Risk-based management framework and exposure definition

**Figure 1:**
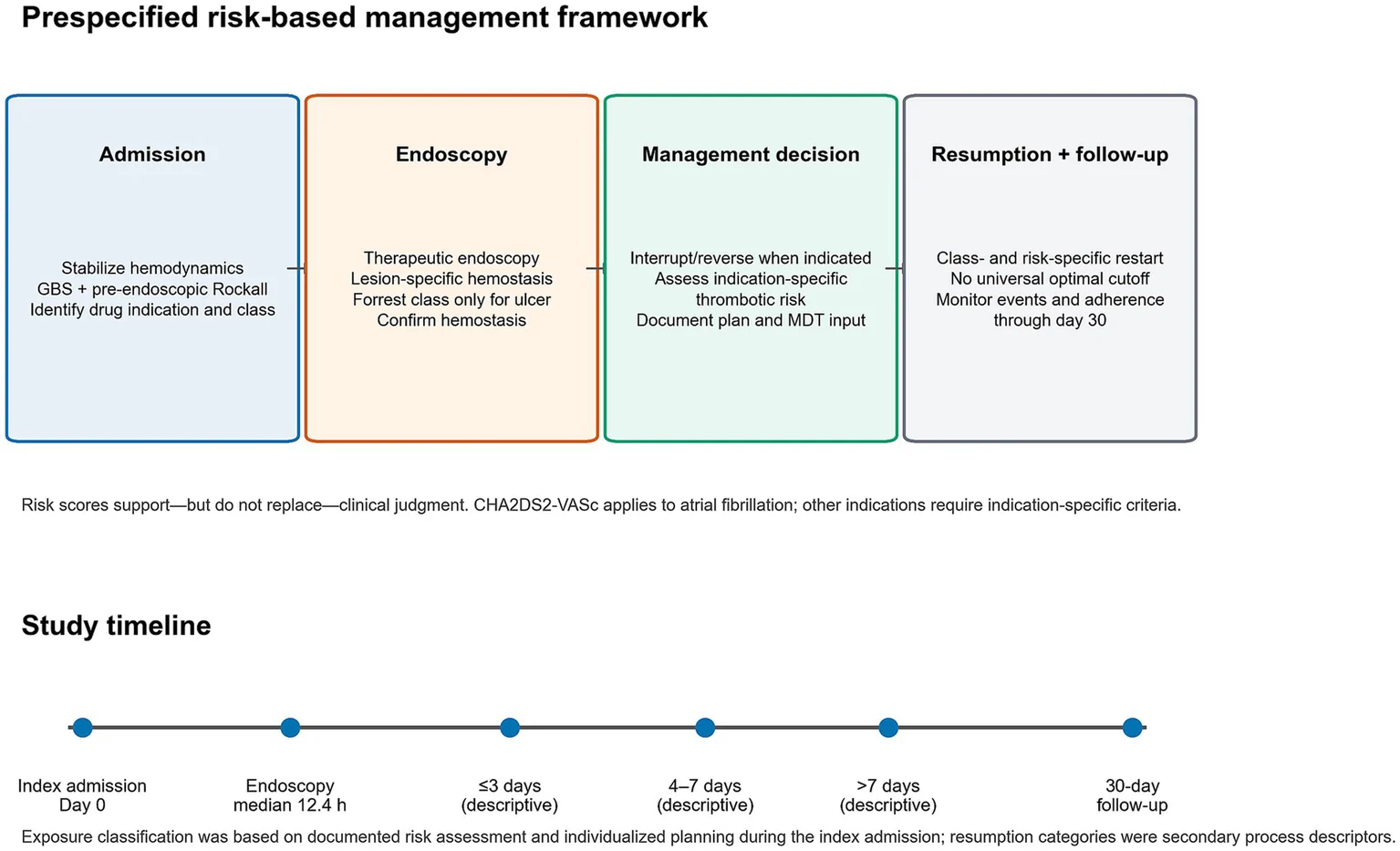
Prespecified risk-based management framework and study timeline. The framework integrates admission bleeding and thrombotic risk, lesion-specific hemostasis, an individualized interruption/reversal/resumption plan, multidisciplinary input, and 30-day monitoring. Timing categories are process descriptors rather than universal recommendations.

The framework comprised four stages (Figure 1 and Supplementary Table S1): admission risk assessment, endoscopic hemostasis, individualized antithrombotic planning, and 30-day surveillance. Admission assessment incorporated hemodynamic status, the Glasgow-Blatchford score, and the pre-endoscopic Rockall score. The Forrest classification was interpreted only as an ulcer-specific endoscopic descriptor. Thrombotic risk was indication-specific: high-risk categories recorded in the dataset were atrial fibrillation with a high CHA2DS2-VASc profile and prior stroke, a mechanical heart valve, venous thromboembolism within 3 months, or recent coronary stenting/acute coronary syndrome.

The primary exposure was documented adherence to this framework, based on a structured chart-review field requiring: (1) documentation of bleeding severity or endoscopic bleeding risk; (2) documentation of the antithrombotic indication or thrombotic-risk category; and (3) an individualized plan for interruption, reversal when indicated, and resumption that was broadly concordant with the framework. Multidisciplinary consultation, actual resumption, and resumption timing were analyzed as downstream process variables and were not mandatory exposure components. Data extraction and exposure classification were independently conducted by two experienced gastroenterologists using a standardized, pre-tested chart-review protocol. Reviewers were blinded to patient 30-day clinical outcomes during the classification of framework adherence. Strategy adherence was defined using strict *a priori* operational criteria requiring complete documentation of: (1) bleeding severity or endoscopic bleeding risk; (2) indication-specific thrombotic risk; and (3) an individualized antithrombotic management plan broadly concordant with institutional pathways. Disagreements between the primary reviewers were resolved through consensus or adjudication by a third senior investigator. Interrater reliability for the primary exposure classification was evaluated in a random 20% sample (*n* = 30), yielding a Cohen’s kappa ( κ ) of 0.86 (95% CI 0.71–1.00), indicating excellent interrater agreement. To reflect real-world clinical documentation practices, we retain the term ‘documented adherence’ throughout the manuscript.

### 2.3 Antithrombotic resumption timing

The interval from hemostasis to first antithrombotic resumption was recorded as no resumption, ≤3 days, 4–7 days, or >7 days. These pragmatic categories were selected to describe early, intermediate, and delayed management decisions in the period when bleeding and thrombosis risks evolve. They are not uniform guideline-concordance categories because recommendations differ by drug class and indication: secondary-prevention aspirin may be resumed when hemostasis is endoscopically confirmed, whereas anticoagulation is generally resumed once bleeding is controlled, preferably within or soon after 7 days according to thrombotic risk (1, 3).

### 2.4 Data sources and variables

Extracted variables included age, sex, body mass index (BMI), smoking and alcohol use, comorbidities, Charlson comorbidity index, antithrombotic class and indication, hemodynamic presentation, laboratory measurements, Glasgow-Blatchford and pre-endoscopic Rockall scores, time to endoscopy, high-risk endoscopic stigmata, hemostatic methods, initial hemostatic success, interruption and reversal, bridging, multidisciplinary consultation, resumption, and 30-day outcomes. CHA2DS2-VASc was applied only to atrial fibrillation; other high-risk categories were defined by the indication-specific criteria above. Endoscopic diagnoses were categorized based on primary visual endoscopic findings into peptic ulcer disease (gastric or duodenal ulcer), erosive gastritis/duodenitis, Mallory-Weiss tears, Dieulafoy lesions, gastric antral vascular ectasia (GAVE) or mucosal angiodysplasia, upper gastrointestinal neoplasms, and other non-variceal mucosal lesions.

### 2.5 Outcomes

The primary outcome was recurrent bleeding within 30 days after successful initial endoscopic hemostasis. Rebleeding required a new clinically documented episode supported by recurrent overt bleeding, hemodynamic deterioration, a secondary hemoglobin decrease, repeat endoscopy, or rescue treatment. Persistent bleeding after unsuccessful initial hemostasis was classified as initial hemostatic failure, not rebleeding. Accordingly, the rebleeding risk set comprised 141 patients with initial hemostatic success.

The key secondary outcome was objectively diagnosed ischemic stroke, myocardial infarction, pulmonary embolism, or deep-vein thrombosis within 30 days. NACE was defined as the occurrence of rebleeding, thromboembolism, or all-cause death within 30 days. Additional outcomes were repeat endoscopy, salvage intervention, readmission, and length of stay.

### 2.6 Data quality and missing data

Before analysis, identifiers, dates, data types, ranges, and cross-field logic were audited. Nine records in which initial hemostasis failed had been coded as rebleeding; these were reclassified as initial hemostatic failure and their recurrent-bleeding dates were cleared. NACE was recomputed from the corrected components. Structural missingness (for example, no rebleeding date when rebleeding did not occur, no bridging agent when bridging was not used, or no resumption date when therapy was not resumed) was not imputed.

Ordinary missingness affected BMI (43/150, 28.7%), albumin (46/150, 30.7%), lactate (54/150, 36.0%), INR (3/150, 2.0%), and duration of antithrombotic use (33/150, 22.0%). Under a missing-at-random assumption, 30 multiply imputed datasets were generated with chained equations and predictive mean matching; observed values were retained and estimates were pooled with Rubin’s rules (13, 14). Primary models used covariates without ordinary missingness and therefore included all eligible patients in each outcome risk set. Multiple-imputation models adding albumin were sensitivity analyses.

### 2.7 Statistical analysis

Distributions were evaluated with histograms, quantile-quantile plots, skewness, and the Shapiro–Wilk test. Approximately symmetric variables are presented as mean (standard deviation); skewed or discrete variables are presented as median [interquartile range]. Categorical variables are shown as number (percentage). Between-group comparisons used Welch’s *t*-test, the Mann–Whitney test, the chi-square test, or Fisher’s exact test as appropriate. Standardized differences are provided for baseline balance; baseline *p*-values were treated descriptively.

Because only 13 rebleeding, 16 thromboembolic, and 29 NACE events occurred, models were prespecified and parsimonious. Firth penalized logistic regression was used to reduce small-sample and separation bias (15). The rebleeding model included documented adherence, shock, and high-risk endoscopic stigmata (*n* = 141). The NACE model included documented adherence, age per 10 years, Charlson index, shock, and high thrombotic risk (*n* = 150). The exploratory thromboembolism model included documented adherence, high thrombotic risk, and Charlson index. Firth regression addresses separation but does not eliminate overfitting; therefore, large estimates and wide intervals were interpreted as directional rather than precise.

We report unadjusted and adjusted odds ratios, 95% confidence intervals, log-odds standard errors, *p*-values, and standardized adjusted risks. Model performance was summarized by the apparent and bootstrap optimism-corrected c-statistic, calibration intercept and slope, Brier score, and Nagelkerke pseudo-*R*^2^. Adjusted *R*^2^ is not defined for logistic regression; pseudo-*R*^2^ was used only as a descriptive goodness-of-fit measure.

Propensity-score matching was considered but not chosen simply on the basis of sample size. One-to-one matching would discard observations and change the target population. Instead, overlap weighting was used as a sensitivity analysis with age, sex, Charlson index, shock, Glasgow-Blatchford score, high thrombotic risk, combination therapy, and high-risk stigmata in the propensity model (16). Balance was assessed with standardized differences and effective sample size.

Resumption timing was not entered into a multivariable outcome model. Seven of the 13 rebleeding events occurred on or before the recorded resumption date, and dates for thromboembolism and death were unavailable. A time-fixed model would therefore be vulnerable to reverse causation and immortal-time bias (17). Timing results are reported as crude, exploratory rates only. Two-sided *p* < 0.05 was considered statistically significant; emphasis was placed on effect estimates and precision.

### 2.8 Ethics statement

The Ethics Committee of The Second People’s Hospital of Linfen approved the study ([2026](003)) and waived informed consent for retrospective use of existing records.

## 3 Results

### 3.1 Participants and baseline characteristics

**Figure 2:**
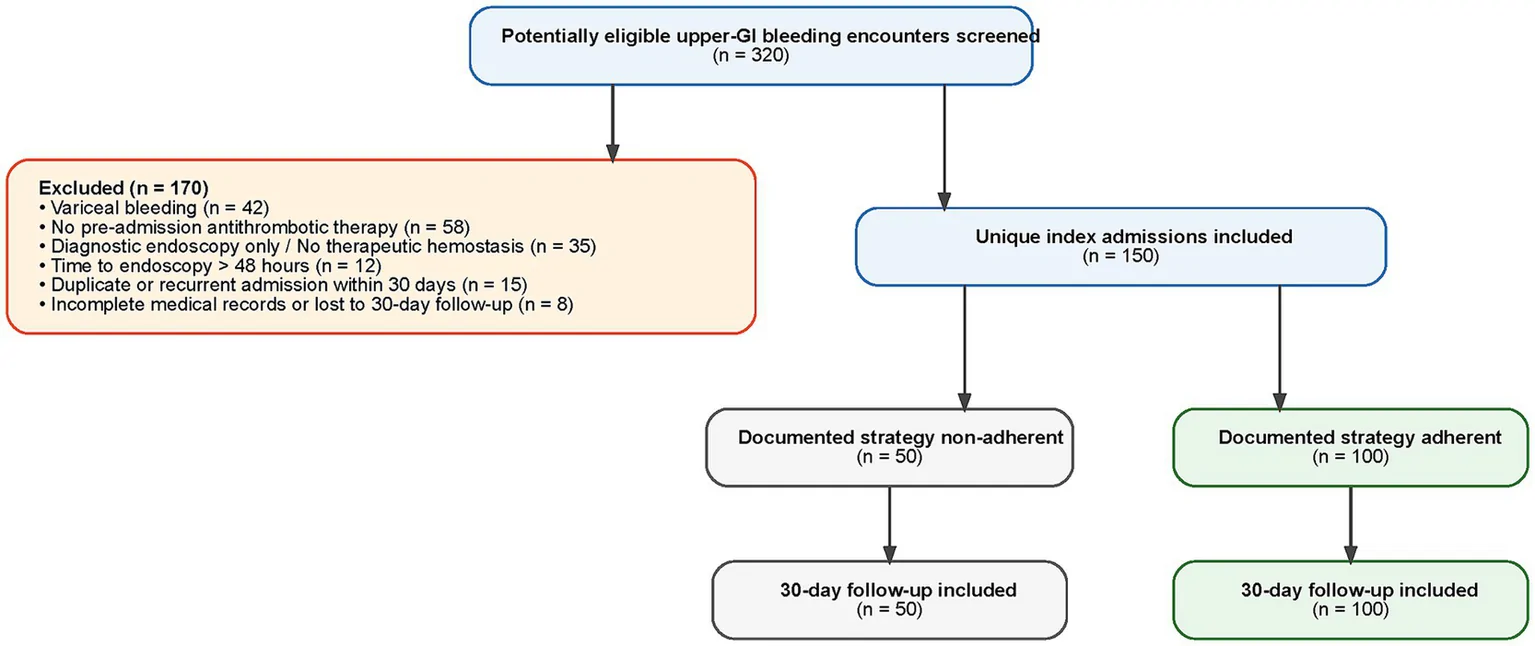
Participant selection flowchart. A total of 320 consecutive patient encounters were screened, 170 were excluded based on predefined criteria, and 150 unique index admissions were analyzed (100 in the documented strategy-adherent group and 50 in the non-adherent group). 30-day rebleeding was evaluated among 141 patients who achieved initial endoscopic hemostatic success.

The corrected cohort comprised 150 unique index admissions: 100 classified as documented strategy-adherent and 50 as non-adherent (Figure 2). Twenty-five admissions occurred in 2021. The mean age was 68.4 (10.7) years and 112 patients (74.7%) were men. High thrombotic risk was present in 28 (18.7%), shock in 37 (24.7%), high-risk endoscopic stigmata in 102 (68.0%), and initial hemostatic success in 141 (94.0%). Baseline standardized differences were generally small; the largest absolute standardized difference in the prespecified propensity covariates was 0.177 (Table 1).

**Table 1:** Baseline characteristics by documented strategy adherence.

Variable	Overall (*n* = 150)	Non-adherent (*n* = 50)	Adherent (*n* = 100)	Missing n	Standardized difference
Age, years	68.4 (10.7)	69.6 (11.0)	67.7 (10.5)	0	−0.18
BMI, kg/m^2^	24.5 (3.4)	24.3 (3.5)	24.6 (3.3)	43	0.11
Charlson comorbidity index	2.0 [2.0, 3.0]	3.0 [2.0, 3.0]	2.0 [1.8, 3.0]	0	−0.10
Hemoglobin on admission, g/L	84.6 (20.9)	85.3 (18.0)	84.2 (22.3)	0	−0.05
INR	1.2 [1.1, 1.6]	1.2 [1.1, 1.8]	1.2 [1.1, 1.5]	3	−0.16
BUN, mmol/L	12.2 [8.7, 15.3]	11.9 [9.1, 14.7]	12.3 [8.6, 15.5]	0	−0.07
Creatinine, μmol/L	88.0 [71.2, 117.5]	92.0 [71.2, 123.5]	85.5 [72.2, 115.8]	0	−0.16
Albumin, g/L	34.8 (4.9)	34.2 (5.2)	35.1 (4.7)	46	0.19
Lactate, mmol/L	1.9 [1.3, 2.7]	2.0 [1.6, 3.1]	1.9 [1.2, 2.6]	54	−0.19
Glasgow-Blatchford score	12.0 [10.0, 14.0]	12.0 [11.0, 13.8]	12.0 [10.0, 14.0]	0	−0.16
Antithrombotic class: Aspirin or P2Y12 inhibitor	45 (30.0%)	13 (26.0%)	32 (32.0%)	0	0.13
Antithrombotic class: Warfarin	8 (5.3%)	4 (8.0%)	4 (4.0%)	0	−0.17
Antithrombotic class: Direct oral anticoagulant	26 (17.3%)	8 (16.0%)	18 (18.0%)	0	0.05
Antithrombotic class: Dual antiplatelet therapy	34 (22.7%)	12 (24.0%)	22 (22.0%)	0	−0.05
Antithrombotic class: Antiplatelet plus anticoagulant	24 (16.0%)	8 (16.0%)	16 (16.0%)	0	0.00
Antithrombotic class: Other	13 (8.7%)	5 (10.0%)	8 (8.0%)	0	−0.07
Pre-endoscopic Rockall score	5.0 [4.0, 6.0]	5.0 [4.0, 6.0]	5.0 [4.0, 6.0]	0	−0.15
Endoscopic diagnosis				0	
Peptic ulcer disease	82 (54.7%)	28 (56.0%)	54 (54.0%)		−0.04
Erosive gastritis/duodenitis	27 (18.0%)	9 (18.0%)	18 (18.0%)		0.00
Mallory-Weiss tear	13 (8.7%)	4 (8.0%)	9 (9.0%)		0.04
Dieulafoy lesion	9 (6.0%)	3 (6.0%)	6 (6.0%)		0.00
GAVE/Angiodysplasia	7 (4.7%)	2 (4.0%)	5 (5.0%)		0.05
Upper GI neoplasm	7 (4.7%)	2 (4.0%)	5 (5.0%)		0.05
Other non-variceal mucosal lesions	5 (3.3%)	2 (4.0%)	3 (3.0%)		−0.05
Time to endoscopy, hours	12.3 (4.7)	11.8 (5.1)	12.6 (4.5)	0	0.16
Length of stay, days	6.0 [5.0, 7.0]	6.0 [5.0, 7.0]	5.5 [5.0, 7.0]	0	−0.13
Male sex	112 (74.7%)	38 (76.0%)	74 (74.0%)	0	−0.05
Current/former smoking	47 (31.3%)	17 (34.0%)	30 (30.0%)	0	−0.09
Alcohol use	35 (23.3%)	10 (20.0%)	25 (25.0%)	0	0.12
Hypertension	69 (46.0%)	23 (46.0%)	46 (46.0%)	0	0.00
Diabetes mellitus	40 (26.7%)	17 (34.0%)	23 (23.0%)	0	−0.25
Coronary artery disease	54 (36.0%)	14 (28.0%)	40 (40.0%)	0	0.26
Atrial fibrillation	33 (22.0%)	11 (22.0%)	22 (22.0%)	0	0.00
Prior stroke/TIA	15 (10.0%)	2 (4.0%)	13 (13.0%)	0	0.33
Chronic kidney disease	15 (10.0%)	5 (10.0%)	10 (10.0%)	0	0.00
Malignancy	15 (10.0%)	7 (14.0%)	8 (8.0%)	0	−0.19
Combination antithrombotic therapy	39 (26.0%)	13 (26.0%)	26 (26.0%)	0	0.00
High thrombotic risk	28 (18.7%)	8 (16.0%)	20 (20.0%)	0	0.10
Shock on admission	37 (24.7%)	10 (20.0%)	27 (27.0%)	0	0.17
High-risk endoscopic stigmata	102 (68.0%)	34 (68.0%)	68 (68.0%)	0	0.00
Combination hemostasis	61 (40.7%)	18 (36.0%)	43 (43.0%)	0	0.14
Initial hemostasis success	141 (94.0%)	45 (90.0%)	96 (96.0%)	0	0.24

Values are mean (SD), median [IQR], or *n* (%). Positive standardized differences indicate higher values in the adherent group.

### 3.2 Management patterns

All patients had antithrombotic therapy temporarily withheld. Reversal was used in 29 (19.3%), bridging in 5 (3.3%), multidisciplinary consultation in 119 (79.3%), and antithrombotic therapy was resumed in 136 (90.7%). Multidisciplinary consultation and resumption were more frequent in the documented-adherence group, but these downstream processes were not components required for exposure classification. Fourteen patients did not resume therapy, 31 resumed within 3 days, 64 within 4–7 days, and 41 after 7 days (Table 2).

**Table 2:** Peri-endoscopic antithrombotic management patterns.

Variable	Overall (*n* = 150)	Non-adherent (*n* = 50)	Adherent (*n* = 100)
Documented bleeding-risk assessment	136 (90.7%)	36 (72.0%)	100 (100.0%)
Documented thrombotic-risk assessment	110 (73.3%)	10 (20.0%)	100 (100.0%)
Antithrombotic therapy withheld	150 (100.0%)	50 (100.0%)	100 (100.0%)
Reversal therapy	29 (19.3%)	9 (18.0%)	20 (20.0%)
Bridging therapy	5 (3.3%)	2 (4.0%)	3 (3.0%)
Multidisciplinary consultation	119 (79.3%)	19 (38.0%)	100 (100.0%)
Antithrombotic therapy resumed	136 (90.7%)	37 (74.0%)	99 (99.0%)
Resumption timing: Not resumed	14 (9.3%)	13 (26.0%)	1 (1.0%)
Resumption timing: ≤3 days	31 (20.7%)	4 (8.0%)	27 (27.0%)
Resumption timing: 4–7 days	64 (42.7%)	7 (14.0%)	57 (57.0%)
Resumption timing: >7 days	41 (27.3%)	26 (52.0%)	15 (15.0%)

Management-process variables were not mandatory components of the exposure definition.

### 3.3 Thirty-day outcomes

Among the 141 patients with successful initial hemostasis, 13 (9.2%) experienced 30-day rebleeding. Rebleeding occurred in 5/96 patients (5.2%) in the documented-adherence group and 8/45 (17.8%) in the non-adherent group (Fisher *p* = 0.026). In the full cohort, thromboembolism occurred in 16/150 (10.7%), all-cause death in 6/150 (4.0%), and NACE in 29/150 (19.3%). NACE occurred in 12/100 adherent patients (12.0%) and 17/50 non-adherent patients (34.0%; *p* = 0.002). Readmission occurred in 9/150 (6.0%) (Table 3).

**Table 3:** Thirty-day clinical outcomes.

Outcome	Overall	Non-adherent	Adherent	Fisher *P*
30-day rebleeding after successful hemostasis	13/141 (9.2%)	8/45 (17.8%)	5/96 (5.2%)	0.026
30-day thromboembolism	16/150 (10.7%)	9/50 (18.0%)	7/100 (7.0%)	0.051
30-day all-cause mortality	6/150 (4.0%)	3/50 (6.0%)	3/100 (3.0%)	0.401
30-day NACE	29/150 (19.3%)	17/50 (34.0%)	12/100 (12.0%)	0.002
Repeat endoscopy	10/150 (6.7%)	5/50 (10.0%)	5/100 (5.0%)	0.302
Salvage intervention	8/150 (5.3%)	2/50 (4.0%)	6/100 (6.0%)	0.719
30-day readmission	9/150 (6.0%)	4/50 (8.0%)	5/100 (5.0%)	0.482

### 3.4 Primary regression analyses

**Figure 3:**
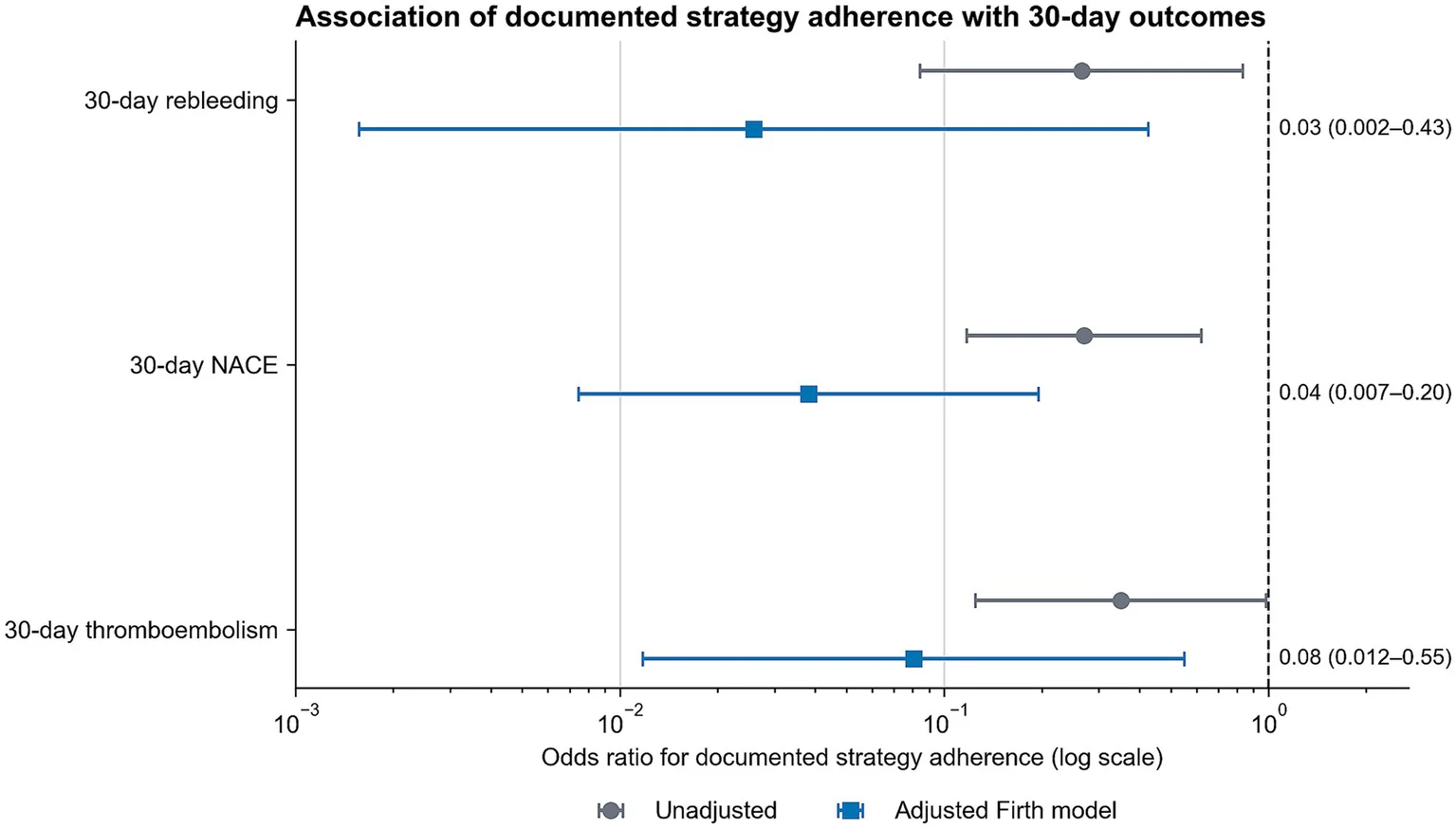
Association of documented strategy adherence with 30-day outcomes. Unadjusted and adjusted Firth odds ratios are shown on a logarithmic scale. Rebleeding analysis was restricted to patients with successful initial hemostasis (*n* = 141); NACE and thromboembolism models included all 150 patients. Exact estimates, standard errors, covariates, and model performance appear in Tables 4–6.

In the parsimonious Firth model, documented adherence was associated with lower rebleeding (aOR 0.03, 95% CI 0.002–0.43; log-odds SE 1.43; *p* = 0.011). The standardized adjusted risks were 5.5% under adherence and 21.2% under non-adherence, corresponding to an adjusted risk difference of −15.7 percentage points (95% bootstrap CI − 23.1 to −9.0). Shock and high-risk stigmata were associated with large, imprecise increases in rebleeding risk (Table 4 and Figure 3).

**Table 4:** Firth logistic regression for 30-day rebleeding after successful initial hemostasis.

Variable	Unadjusted OR (95% CI)	SE	*P*	Adjusted OR (95% CI)	SE	*P*
Documented strategy adherence (yes vs. no)	0.27 (0.08–0.83)	0.59	0.023	0.03 (0.002–0.43)	1.43	0.011
Shock on admission (yes vs. no)	44.11 (7.52–258.76)	0.90	<0.001	335.69 (16.01–7037.07)	1.55	<0.001
High-risk endoscopic stigmata (yes vs. no)	4.86 (0.85–27.89)	0.89	0.076	22.17 (1.24–395.11)	1.47	0.035

Rebleeding model: shock and high-risk endoscopic stigmata; *n* = 141, 13 events. Apparent/optimism-corrected c-statistic 0.971/0.967; calibration intercept 0.05, slope 1.12; Brier score 0.033; Nagelkerke pseudo-R^2^ 0.714. SE is the standard error of the log odds ratio.

Documented adherence was also associated with lower NACE (aOR 0.04, 95% CI 0.007–0.20; SE 0.83; *p* < 0.001). Standardized adjusted risks were 12.1 and 39.5%, respectively, for a risk difference of −27.4 percentage points (95% CI − 34.2 to −17.1). The exploratory thromboembolism model produced a concordant association (aOR 0.08, 95% CI 0.012–0.55), but only 16 events were available (Tables 5, 6). These large estimates reflected sparse cells and near-complete separation and should not be interpreted as precise effect sizes.

**Table 5:** Firth logistic regression for 30-day NACE.

Variable	Unadjusted OR (95% CI)	SE	*P*	Adjusted OR (95% CI)	SE	*P*
Documented strategy adherence (yes vs. no)	0.27 (0.12–0.62)	0.43	0.002	0.04 (0.007–0.20)	0.83	<0.001
Age (per 10 years)	1.00 (0.69–1.46)	0.19	0.995	0.89 (0.49–1.61)	0.30	0.704
Charlson comorbidity index (per point)	1.20 (0.92–1.57)	0.14	0.175	1.58 (1.05–2.39)	0.21	0.029
Shock on admission (yes vs. no)	5.71 (2.41–13.57)	0.44	<0.001	50.98 (9.06–286.96)	0.88	<0.001
High thrombotic risk (yes vs. no)	10.71 (4.19–27.38)	0.48	<0.001	144.68 (19.19–1090.96)	1.03	<0.001

NACE model: age, Charlson index, shock, and high thrombotic risk; *n* = 150, 29 events. Apparent/optimism-corrected c-statistic 0.957/0.941; calibration intercept −0.01, slope 1.06; Brier score 0.065; Nagelkerke pseudo-*R*^2^ 0.672. SE is the standard error of the log odds ratio.

**Table 6:** Exploratory Firth logistic regression for 30-day thromboembolism.

Variable	Unadjusted OR (95% CI)	SE	*P*	Adjusted OR (95% CI)	SE	*P*
Documented strategy adherence (yes vs. no)	0.35 (0.12–0.98)	0.53	0.046	0.08 (0.01–0.55)	0.98	0.010
High thrombotic risk (yes vs. no)	48.20 (11.20–207.45)	0.74	<0.001	217.01 (23.47–2006.38)	1.13	<0.001
Charlson comorbidity index (per point)	1.29 (0.93–1.80)	0.17	0.126	1.95 (1.14–3.32)	0.27	0.014

Thromboembolism model: high thrombotic risk and Charlson index; *n* = 150, 16 events. Apparent/optimism-corrected c-statistic 0.965/0.959; calibration intercept 0.01, slope 1.05; Brier score 0.041; Nagelkerke pseudo-*R*^2^ 0.672. SE is the standard error of the log odds ratio.

### 3.5 Model performance and sensitivity analyses

The apparent c-statistic was 0.971 for rebleeding and 0.957 for NACE; bootstrap optimism-corrected values were 0.967 and 0.941. Calibration slopes were 1.12 and 1.06, Brier scores were 0.033 and 0.065, and Nagelkerke pseudo-*R*^2^ values were 0.714 and 0.672, respectively. These unusually high in-sample metrics arose in a small dataset with near separation and do not establish transportable predictive performance (Supplementary Table S2 and Supplementary Figure S1).

Multiple-imputation models that added albumin were directionally concordant: the pooled adherence OR was 0.03 (95% CI 0.002–0.42) for rebleeding and 0.04 (95% CI 0.008–0.21) for NACE. Propensity scores ranged from 0.452 to 0.829. Overlap weighting reduced the maximum absolute standardized difference from 0.177 to <0.001, with an effective sample size of 130.1. The overlap-weighted OR was 0.18 (95% CI 0.06–0.61) for rebleeding and 0.20 (95% CI 0.08–0.46) for NACE. The attenuation relative to outcome regression reinforces the need for cautious interpretation.

### 3.6 Exploratory resumption timing

**Figure 4:**
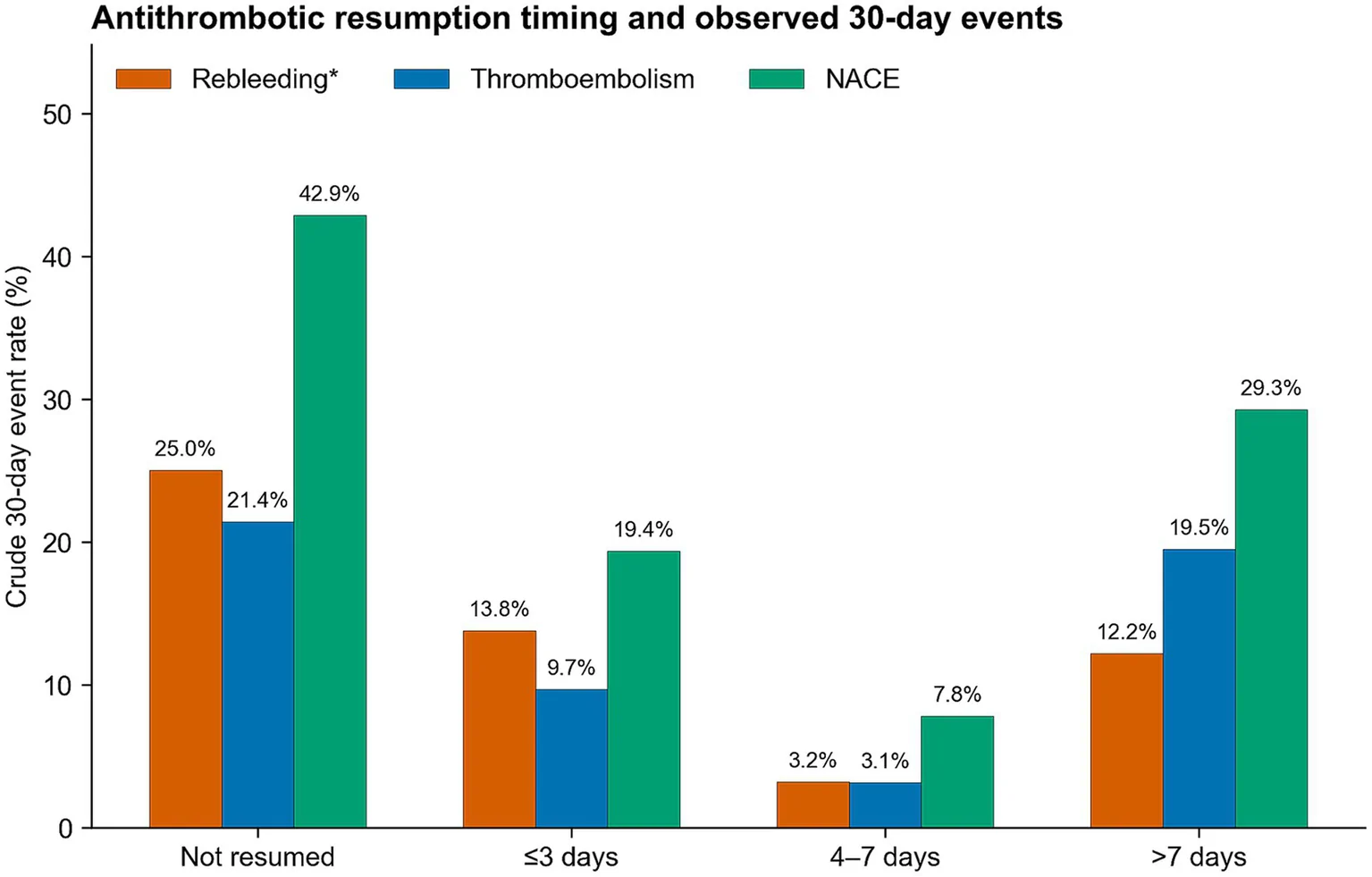
Antithrombotic resumption timing and observed 30-day events. Bars show crude rebleeding, thromboembolism, and NACE rates. Rebleeding denominators include only patients with successful initial hemostasis. Seven of 13 rebleeding events occurred on or before the recorded resumption date; the figure is descriptive and does not identify an optimal restart interval.

Crude rebleeding rates after successful hemostasis were 25.0% (2/8) with no resumption, 13.8% (4/29) with resumption within 3 days, 3.2% (2/63) at 4–7 days, and 12.2% (5/41) after 7 days. Corresponding thromboembolic-event rates in the full timing groups were 21.4, 9.7, 3.1, and 19.5% (Figure 4 and Supplementary Table S3). Because seven rebleeding events occurred on or before the recorded resumption date, these crude differences cannot identify an optimal interval and were not adjusted.

## 4 Discussion

In this corrected retrospective cohort, documented adherence to a risk-based antithrombotic management framework was associated with fewer 30-day rebleeding events and NACE. The association was present in parsimonious Firth models, multiple-imputation sensitivity models, and propensity-score overlap-weighted analyses. The central clinical implication is not that one documentation label or restart day causes better outcomes. Rather, the findings support prospective evaluation of structured, indication-specific assessment that integrates bleeding control, thrombotic risk, endoscopic findings, and follow-up.

The effect estimates from outcome regression were extremely large. Sparse cells and near-complete separation were visible in the data: few events occurred among adherent patients without shock or high thrombotic risk. Firth penalization yields finite estimates but cannot create information that is absent. The more moderate overlap-weighted estimates, wide confidence intervals, and high apparent discrimination all point to the same conclusion: direction is more credible than magnitude, and external validation is required.

The exposure must also be interpreted as documented adherence. Patients with a complete risk assessment and individualized plan may have been treated by clinicians or teams with greater expertise, better access to consultation, more intensive nursing, stronger discharge planning, or more complete follow-up. These unmeasured attributes can influence both documentation and outcomes. Multivariable regression and propensity weighting adjust only measured factors and cannot eliminate confounding by indication, documentation quality, or institutional workflow.

The timing findings were deliberately downgraded to descriptive evidence. Although the observed 4–7-day group had the lowest crude event rates, later classification requires patients to remain alive and event-free long enough to enter that group. Early deterioration may also determine non-resumption. Because more than half of rebleeding events occurred on or before the recorded restart date and dates for thrombotic events and death were unavailable, a conventional logistic timing model would be biased. Guidelines likewise do not support one threshold across all drugs and indications; individualized multidisciplinary assessment is more defensible than a universal 4–7-day rule (1, 3).

The study has several strengths. It separates persistent failure of initial hemostasis from recurrent bleeding, uses an objective index-admission rule, retains all eligible observations in the main outcome models, distinguishes structural from ordinary missingness, and triangulates outcome regression with multiple imputation and overlap weighting. Absolute risks, standardized differences, model-performance measures, and full model standard errors are reported. The focus on 30-day follow-up is clinically relevant because early recurrent care after UGIB remains common (10).

Limitations are substantial. This was a single-center retrospective study with only 13 rebleeding and 29 NACE events. Exposure classification depended on documentation and could not be independently re-adjudicated from the analytic file; reviewer blinding and interrater agreement were unavailable. Residual confounding, differential follow-up, and outcome misclassification remain possible. The missing-at-random assumption for multiple imputation is untestable. The screening extract did not contain reason-specific exclusion counts, and the numeric endoscopic-diagnosis codes lacked a verified dictionary. Four post-discharge rebleeding records had no coded readmission and require source-record confirmation. Finally, the timing analysis was vulnerable to immortal-time and reverse-causation biases and lacked event dates needed for time-dependent modeling.

## 5 Conclusion

Documented adherence to a risk-based antithrombotic management framework was associated with lower 30-day rebleeding and NACE after therapeutic endoscopy for NVUGIB. The findings are hypothesis-generating, not causal, and the magnitude of association is uncertain because of sparse data, documentation-dependent exposure classification, and residual confounding. Individualized multidisciplinary assessment appears more important than adherence to any single predefined resumption threshold.

## Data Availability

The raw data supporting the conclusions of this article will be made available by the authors, without undue reservation.

## Supplementary material

SUPPLEMENTARY FIGURE S1 Calibration of the parsimonious Firth models. Points show observed and mean predicted risks across fifths of predicted risk, with Wilson confidence intervals. Apparent calibration is reported for descriptive internal assessment only.
